# Vertical Distribution of Total Mercury and Mercury Methylation in a Landfill Site in Japan

**DOI:** 10.3390/ijerph15061252

**Published:** 2018-06-13

**Authors:** Jing Yang, Masaki Takaoka, Akira Sano, Akito Matsuyama, Ryuji Yanase

**Affiliations:** 1 Department of Environmental Engineering, Graduate School of Engineering, Kyoto University, C-kluster, Kyotodaigakukatsura, Nishikyo-ku, Kyoto 6158540, Japan; yang.jing.53s@st.kyoto-u.ac.jp; 2 Graduate School of Global Environmental Studies, Kyoto University, C-kluster, Kyotodaigakukatsura, Nishikyo-ku, Kyoto 6158540, Japan; sano.akira.2a@kyoto-u.ac.jp; 3 National Institute for Minamata Disease, 4058-18 Hama, Minamata-City, Kumamoto 8670008, Japan; matsuyam@nimd.go.jp; 4 Environmental Protection Center, Fukuoka University, 8-19-1 Nanakuma, Jonan-ku, Fukuoka 8140180, Japan; ryanase@fukuoka-u.ac.jp

**Keywords:** mercury, landfill, core sampling, hgcA, merA, merB

## Abstract

Mercury is a neurotoxin, with certain organic forms of the element being particularly harmful to humans. The Minamata Convention was adopted to reduce the intentional use and emission of mercury. Because mercury is an element, it cannot be decomposed. Mercury-containing products and mercury used for various processes will eventually enter the waste stream, and landfill sites will become a mercury sink. While landfill sites can be a source of mercury pollution, the behavior of mercury in solid waste within a landfill site is still not fully understood. The purpose of this study was to determine the depth profile of mercury, the levels of methyl mercury (MeHg), and the factors controlling methylation in an old landfill site that received waste for over 30 years. Three sampling cores were selected, and boring sampling was conducted to a maximum depth of 18 m, which reached the bottom layer of the landfill. Total mercury (THg) and MeHg were measured in the samples to determine the characteristics of mercury at different depths. Bacterial species were identified by 16S rRNA amplification and sequencing, because the methylation process is promoted by a series of genes. It was found that the THg concentration was 19–975 ng/g, with a geometric mean of 298 ng/g, which was slightly less than the 400 ng/g concentration recorded 30 years previously. In some samples, MeHg accounted for up to 15–20% of THg, which is far greater than the general level in soils and sediments, although the source of MeHg was unclear. The genetic data indicated that hgcA was present mostly in the upper and lower layers of the three cores, merA was almost as much as hgcA, while the level of merB was hundreds of times less than those of the other two genes. A significant correlation was found between THg and MeHg, as well as between MeHg and MeHg/THg. In addition, a negative correlation was found between THg and merA. The coexistence of the three genes indicated that both methylation and demethylation processes could occur, but the lack of merB was a barrier for demethylation.

## 1. Introduction

Mercury, as one of the most toxic pollutants in the earth’s biogeochemical system and the human ecosystem, has become a global environmental concern. Due to its persistent and bio-accumulative properties, mercury, especially methyl mercury, is a potent neurotoxin that can affect the health of wildlife and humans. One of the most remarkable physical properties of mercury and some of its compounds is high volatility, which leads to the potential for long-range transport [1]. Mercury occurs naturally in the environment, but the levels of mercury in the atmosphere and oceans have increased due to human activities, such as mining [2,3], fossil fuel combustion [4], and the chlorine alkali industry [5]. Landfill sites are complex systems, with layers of various depths under aerobic or anaerobic conditions. The methylation process is greater under anaerobic than aerobic conditions [6], and landfill sites are a significant emission source of total gaseous mercury, as well as methyl mercury (MeHg) [7]. Hence, the behavior of mercury in landfills needs to be investigated.

In response to the Minamata Convention, which considered the problem of global mercury transport and the human health impacts of mercury pollution [8], the control of mercury emissions will likely be difficult because of the lag time in natural systems, meaning that a reduction in emissions will not have an immediate impact on exposure [9]. The influence of previously emitted anthropogenic mercury will last for a long time, and mercury products currently in existence will eventually accumulate in landfill sites following their disposal. 

As a pervasive global pollutant, mercury especially in the form of MeHg bioaccumulates in the food chain and is highly toxic to human beings. The organic forms of mercury differ substantially from the inorganic forms, which are derived from anthropogenic emissions and subsequent atmosphere deposition, while organic forms of mercury are produced in the environment following the transport of inorganic mercury. It has been reported that in anaerobic environments, micro-organisms predominantly generate MeHg from inorganic forms of mercury [10]. Mercury methylation is promoted by enzyme catalysis, during which a methyl group is transferred to inorganic mercury from the methylated hgcA protein [11]. Sulfate-reducing bacteria have been identified as primary producers of MeHg in the environment, with iron-reducing bacteria and methanogens also being involved in this process [12]. 

In general, the mercury pollution potential of a landfill is dependent on two factors: gaseous emissions and leachate. The airborne emission of mercury from landfills has been widely reported. As a mercury sink, landfills act as a source of mercury from both landfill gas [13,14,15,16,17] and leachate [18]. Therefore, if the landfill acts as a methylation and demethylation reactor, the behavior of mercury in the landfill will have a strong influence on the generation of both MeHg and elemental mercury. A study in China reported on the mercury distribution within the top layer of soil (0–15 cm) in the largest active landfill in Asia [19], while another study surveyed mercury in a municipal solid-waste landfill in Florida [20]. Samples were collected from cores at depths of approximately 3–12 m, but no information was provided regarding the vertical distribution of methylation. Generally only the top layer of soil has been considered in assessments of mercury-contaminated sites [21,22,23]. As an artificial contaminated site, a landfill is a complex environment, and its assessment requires a three-dimensional analysis. In recent years, landfill sites as Hg emission source have caused more concern, but a gap of knowledge still remains with respect to the mechanisms of the methylation process [24,25]. Up to now, published studies regarding the methylation and demethylation processes in landfills have rarely been seen. 

To provide a better understanding of the processes affecting mercury in landfills, we conducted vertical boring sampling in three locations at a landfill site in Japan. We analyzed the levels of total mercury (THg), MeHg, and three bacterial genes (hgcA, merA, and merB), which play key roles in controlling bacterial methylation and demethylation processes in a closed landfill site. The aim was to further determine the changes in mercury speciation in landfill sites and the bacterial genes that control methylation and demethylation, which would ultimately provide a better explanation of mercury release and emission from landfill sites.

## 2. Materials and Methods

### 2.1. Sample Collection

The study was conducted in January 2015 at a landfill site in Japan. Samples were collected from closed areas in the landfill site, which was constructed in 1965 and began operating in 1978. The site accepts approximately 2.4–2.7 × 10^4^ tons of municipal solid waste per year, and the total amount of waste on-site at the time of the study was approximately 4.48 × 10^5^ tons. This landfill site received waste collected from citizens who lived in a city, as well as a portion of incinerator bottom ash. The landfilled waste consisted of 34.0% incineration ash, 23.8% debris or earth dug out of construction site, 16.2% foundry sand, 12.8% biomass waste, 5.2% metal and 2.2% plastics. The landfill was designed with clay layers at the surface and in the middle of the sampling cores, clay was constructed between each 3-m to 4-m layer of waste. A boring machine was used for the core sampling, and cylindrical samples (8 cm diameter) were collected from three locations designated as Cores 1, 2, and 3 (16, 18, and 11 m deep, respectively). The waste samples collected from each borehole were placed in four rows inside 1 m long wooden cases. One cylindrical sample (length × diameter: 10 × 8 cm) was selected as a representative of each 1 m core sample and was stored in a vacuum bag inside an anaerobic pouch to prevent contact with oxygen. It was placed in a refrigerator at 5 °C until it was processed on site. All operations referred to above were conducted on site. Sample pretreatment in the laboratory involved the separation and characterization of waste components. Non-degradable and slowly degrading materials such as stones, plastic bags, and ceramics in the samples, which occupied 15–30% of the total weight, were removed, and the remaining materials were passed through a 5 mm sieve. Any changes in soil characteristics, such as color and texture, were monitored, and the compositions of waste summarized in Table S1 (Supplementary Materials). Because classification was not carried out completely at the landfilling time, we found glass, wood, metal can, dry batteries in the core samples. Over 40 years’ degradation, food waste had turned to humus. After transportation back to the laboratory, the central portion of the cylindrical samples was separated into culture dishes for gene analysis and small vacuum bags for mercury analysis.

### 2.2. Analysis of THg and MeHg Concentrations

The THg concentration of each core sample was determined by a mercury analyzer (MA-2000, Nippon Instruments, Tokyo, Japan), which enabled the mercury content to be measured at different depths based on Japanese industrial standard M8801. The samples used were sieved and ground manually. Approximately 50 mg of samples were used in each measurement, with the addition of two different chemical additives, and each analysis was performed in triplicate. Mercury in the samples was vaporized in the heater to free the mercury vapor in the gas generated, which was collected by a mercury collection agent (a gold-coated diatomite particle support) in the form of gold amalgam. The mercury collection agent was then heated to 850 °C to release the atomic mercury, which was detected using the cold atomic absorption method [26] at a wavelength of 253.7 nm in an absorption cell. 

In the case of MeHg, two samples with a high THg content in each sampling core were chosen as representative samples. The analysis of MeHg was conducted using dithizone extraction and electron capture detector (ECD) gas chromatography using ECD gas chromatography produced by YANACO Co., Ltd., (Kyoto, Japan, Model G3800) under the condition of a 3.0 mm–0.75 m glass column packed with Hg-20 A on Uniport HP (AW-DMCS, 60–80 mesh, GL Sciences Co., Ltd., Tokyo, Japan), following the method of Akagi [27] as modified by Matsuyama [28]. Briefly, approximately 0.2 g of the sieved sample was placed into a 50 mL centrifuge tube, and 10 mL 1 N KOH/ethanol was added. Then, the sample was ground using a glass stick, followed by shaking to dissolve the MeHg. MeHg in the mixture was then extracted by addition of purified 0.01% dithizone–toluene solution (5 mL). After cleanup of the dithizone–toluene extract, the sample solution was analyzed by ECD gas chromatography.

### 2.3. Gene Analysis

Using an Extrap Soil DNA Kit Plus ver. 2 (Nittetsu Sumikin Kankyo, Inc., Ibaraki, Japan), total microbial DNA was extracted from a 0.5 g sub-sample of approximately half the total number of samples. The diluted DNA in each sample was subjected to reverse-transcription quantitative polymerase chain reaction (RT-qPCR) to determine the abundance of three particular genes: hgcA (mercury methylation), merA (mercury reduction), and merB (mercury demethylation). HgcA has previously been identified as being involved in mercury methylation (Parks et al., 2013). MerB catalyzes protonolysis of the carbon–mercury bond, resulting in a reduced carbon compound and inorganic ionic mercury. MerA reduces ionic mercury to elemental mercury (Benison et al., 2004), which is the final step in the production of Hg^0^. The hgcA, merA, and merB genes were quantified using primer pairs reported previously [29,30,31]. RT-qPCR was performed using SYBR green I on the Rotor-Gene Q (QIAGEN, Holland). RT-qPCR for hgcA, merA, and merB was performed. The levels of 16S ribosomal RNA (rRNA) gene, which is a highly abundant bacterial gene, were quantified for comparison with the levels of the genes evaluated for each sample using the same PCR machine.

## 3. Results and Discussion

### 3.1. THg

Perforation sampling was started from the top soil layer of the landfill surface, ending at the bottom of the landfill. Depending on the stratification of the cover soil at the top of the landfill, waste, the bottom clay layer, and other layers, the properties of the samples were very diverse. The waste is located between the soil cover, and the clay and is mixed in with soil.

**Figure 1:**
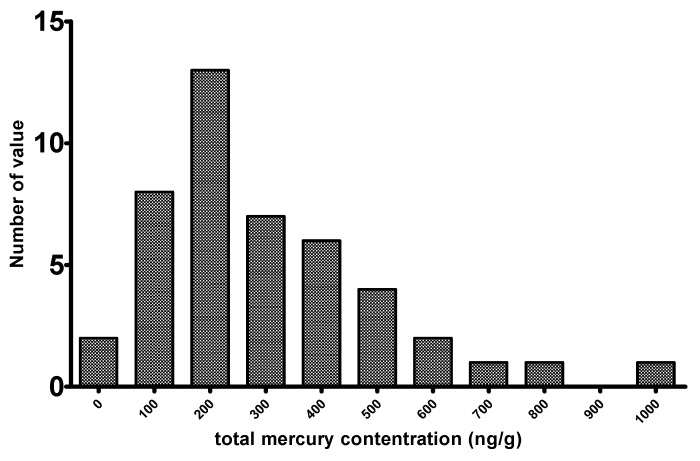
Frequency distribution of THg concentrations in core samples.

THg was measured in 44 core samples, and the mercury concentrations ranged from 19 to 975 ng/g, with a geometric mean of 299 ng/g (sd: 203). As listed in Table 1, the mean THg concentrations in Cores 1, 2, and 3 were similar and ranged from 200 to 400 ng/g. Compared to the survey report, in which THg was reported as 400, on average, at this site, the average content of THg was slightly reduced. However, a large proportion of THg remained in the landfill site, and the first decade is typically the most active period of chemical processes in a landfill site. We suggest that this landfill site has been approaching the steady stage recently, and a landfilling time of several decades did not lead to high Hg losses. Figure 1 shows the skewed frequency distribution of the THg concentrations of these 44 samples; most samples had a concentration of less than 500 ng/g, with only one sample having a concentration at around the 1000 ng/g level.

**Figure 2:**
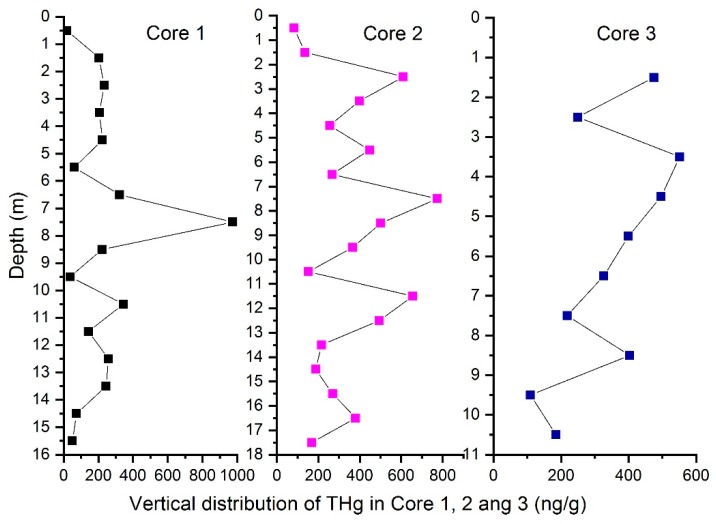
Vertical distribution of total mercury in solid samples in core 1, 2 and 3 (ng/g).

The mean THg concentrations at the three cores were 226, 353, and 316 ng/g, respectively. Figure 2 shows the vertical distribution of the THg concentrations in cores 1, 2 and 3; the concentrations were spatially uneven in the three cores, and the highest concentrations were found in the 8-m-depth layer of Cores 1 and 2. The minimum concentration was 19 ng/g in a sample obtained close to the surface layer in Core 1, while the lowest concentrations in Cores 2 and 3 were also in the top layer, indicating that little mercury in the waste was transported to the upper cover soil layers. The THg concentrations were comparable to those measured in other studies in landfills, which range from 32.8 to 16,800 ng/g, with a geometric mean of 178 ng/g, in U.S. sites [20]. Usually, the THg concentration in the top layers of soil from contaminated sites has a higher content, as reported previously, for example, 0.5–3000 × 103 ng/g [21], 2 × 103 ng/g [22], and 6.3–8600 × 103 ng/g [23]. However, in this case, the top soil layer had a THg content of less than 100 ng/g, while higher levels were detected in deeper samples. It can be seen from Figure 2 that the core samples were separated into several sections depending on the THg content. The top layer and bottom cover had the lowest Hg contents in all three cores, with higher levels of THg measured in the waste layer between them. THg distribution in a landfill depends on the characteristics of the original waste, and the results obtained in this study indicate that most of the THg remained in the landfill, rather than being emitted in leachate. The separation of THg among the different sections of the landfill also suggested that Hg remained at the location where it was initially disposed. If a large amount of mercury is lost from a landfill, the THg content should display a tendency to decrease or increase vertically. However, the THg distribution in each sampling core displayed a clear peak, as shown in Figure 2. Studies of the behavior of Hg released from used batteries in model landfills over a 20-year period reached a similar conclusion, with Hg found to remain mostly in the same landfill layer [32]. 

### 3.2. MeHg

**Figure 3:**
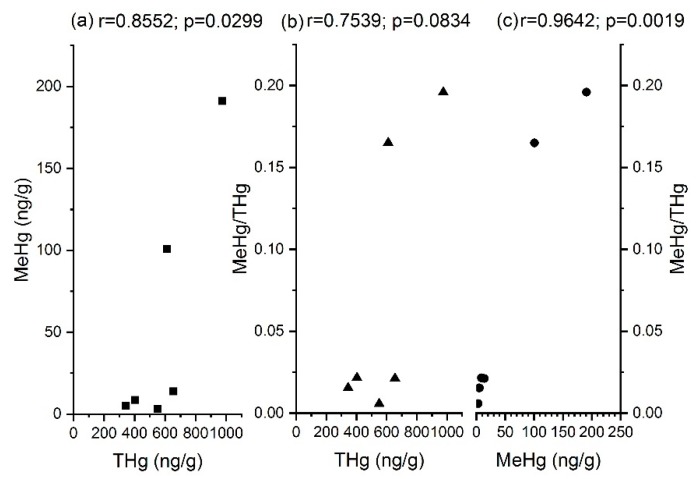
Correlation between THg and MeHg: (**a**) Relation between THg and MeHg; (**b**) Relation between THg and MeHg/THg; (**c**) Relation between MeHg and MeHg/THg.

Two samples from each core were selected for MeHg measurements, and the results are presented in Table 2. In the 8-m-layer sample from Core 1 and the 3-m-layer sample from Core 2, MeHg accounted for 19.6% and 16.5% of the THg content, respectively. In the other samples MeHg accounted for less than 3% of the THg content. Van Faassen [33] reported that the accumulation rate of MeHg in sludge and sediment was, in most cases, 1%, and only in one sample did it reach 5%. Another study reported that MeHg accounted for an average of 0.77% of the THg in sediments [34]. Extremely high proportions of MeHg in soil or waste samples have rarely been reported; in contrast, MeHg has been shown to account for 3–12% of THg in marine mammals [35]. A significant correlation between THg and MeHg was observed (r = 0.8553, *p* < 0.05), as shown in Figure 3a, with similar relationships of waste samples rarely reported; however, a positive correlation was reported of surface water and soils [36]. Because there were some interferences in the MeHg measurements in waste samples, the MeHg data is limited in this case. The positive correlation indicates a potential influence of THg on MeHg, but further research is required at more landfill sites. 

Despite the high accumulation rate and significant correlation, no clear evidence was found that methylation had occurred in the landfill, or that the stability of the Hg disposed of in the landfill was altered over time, because the source of the MeHg was not determined. While cysteine, a commonly-used food additive, has been proved to be essential for Hg methylation [37], which possibly might lead to a promotion of Hg methylation in a landfill that contains food residuals. As shown in Figure 3b, THg positively correlates with the ratio of MeHg to THg (r = 0.7539; *p* > 0.05), although it is not significant. However, the opposite correlation was found in sediment samples [34].

### 3.3. Analysis of the hgcA, merA, and merB Genes with Regard to Hg Speciation

We selected 70% of all samples for the gene analysis. The common 16S rRNA gene and the hgcA, merA, and merB genes were amplified by RT-qPCR. As an indicator of the abundance of bacteria, 16S rRNA gene sequences have demonstrated a huge diversity in bacterial communities [38]. The 16S rRNA gene copy numbers in dry soil is at the magnitude of 10^8^ to 10^9^ [39], while in this case, the 16S rRNA copy numbers ranged from 10^6^ to 10^9^, as shown in Table 3. In the top and bottom soil layers, the 16S rRNA copy numbers were at the magnitude of 10^8^, which is similar to that of normal soil. During the extraction of DNA from waste samples, we faced difficulties in isolating microbial DNA, which is commonly observed in environmental samples [40,41].

The fate of mercury in a landfill site is a dynamic process that includes formation (methylation) and degradation (demethylation), which is the opposite of oxidizing inorganic mercury to organic forms. The methylation process occurs via the activity of hgcA [37,42], while demethylation, which generally refers to the cleavage of the H_3_C–Hg bond, as well as the reduction of ionic mercury to elemental mercury [43], is controlled by bacterial enzymes, which are encoded by mercury resistance operons. Roles of the mercuric reductase enzyme encoded by merA and organomercurial lyase enzyme encoded by merB in this process have been identified [44]. The merA and merB enzymes play critical roles in the transportation and transformation of mercury in the environment [45]. This process has been well examined in aerobic environments, but merA activity in anaerobic environments remains unclear. 

**Figure 4:**
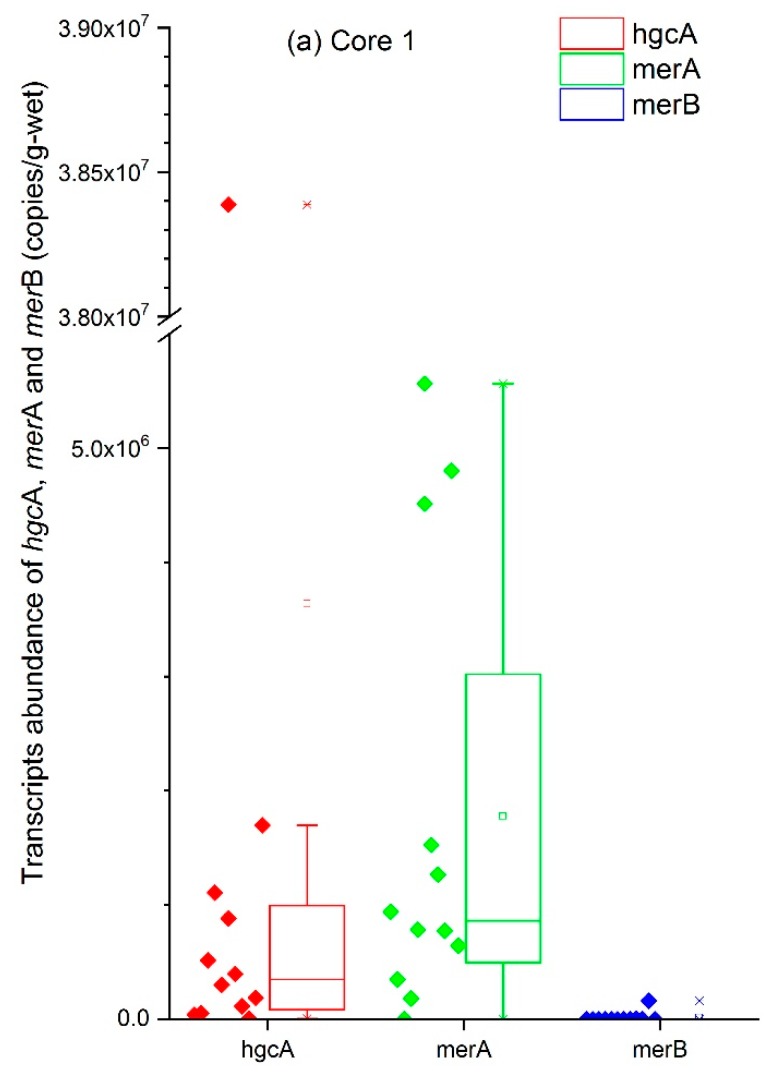

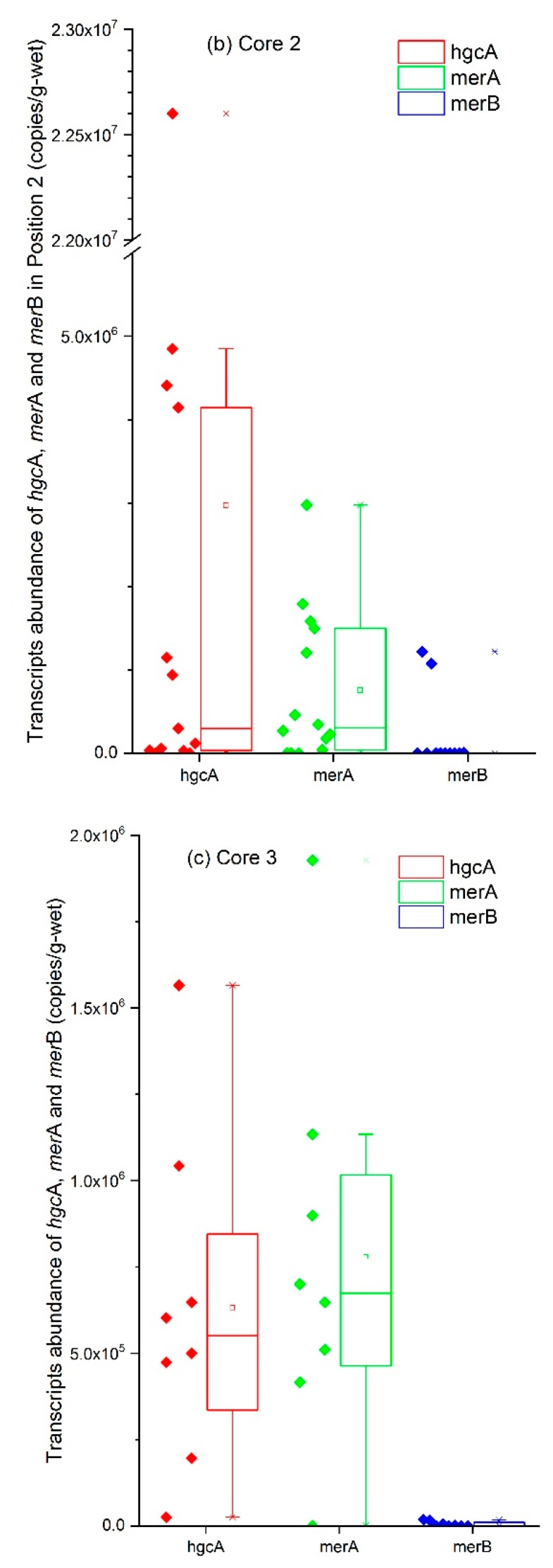
Whisker and box plot representation of the hgcA, merA and merB genes in three sampling cores. (**a**) box plot of hgcA, merA and merB in Core 1; (**b**) box plot of hgcA, merA and merB in Core 2; (**c**) box plot of hgcA, merA and merB in Core 3.

The hgcA gene plays an essential role in mercury methylation and has been found in abundance in wetland soils [46,47], paddy soils [29], inundated and non-inundated soil [48], and the aquatic environment downstream of a chlor-alkali plant [43]. In this study, we detected hgcA in the core samples from a landfill site, as shown in Figure 4. The copy numbers of each gene, as well as the expression levels relative to those of 16S rRNA, as determined by RT-qPCR, showed a distinct distribution in expression among the three cores. 

The transcript of hgcA was the most abundant functional transcript among the measured ones in Core 1 and 2 (Figure 4). The higher mean level of hgcA in each core indicates the landfill offered a better living condition for it than merA and merB. An outlier detection appeared in both Core 1 and 2 that the two samples (No. 1–1 and No. 2–12) showed the highest ratio of hgcA to 16S rRNA and relatively high ratios of merA and merB (Table S1 in Supplement Materials). HgcA performed more actively in the upper layers, which indicates that waste inhibited the growth of bacteria with hgcA. No correlation was found between the activity of the hgcA gene and THg, MeHg content in samples or in the ratio between MeHg and THg (Table 4). The bacterial ability to methylate Hg has been demonstrated to depend on the presence of the hgcAB gene cluster in the laboratory [42,49]; however, the concentration of MeHg in environmental samples is the result of multiple factors, including the balance of Hg methylation, MeHg demethylation and Hg^2+^ reduction to Hg. Specifically, samples in landfills have another possibility to gain MeHg, for instance, from the original waste which might contain some food residual. This could explain the noncommittal correlation between the gene expression level and the MeHg concentration. Similar poor correlation was also observed in sediment samples [43] and even in lab study [50]. This indicates that environmental factors had more influence than did gene expression levels. Hg methylation cannot be explained by the abundance of hgcA gene alone, but also the bioavailability of Hg to the bacteria [51].

The bacterial resistance of MeHg is determined by the merA and merB genes. These enzymes act sequentially, such that merB cleaves the C-Hg bond of MeHg to CH_4_ and mercuric ion, while merA reduces mercuric ion to metal mercury [52]. As shown in Figure 4, merA appeared as the same average level with hgcA and existed extensively in most samples. In contrast, merB gene was detected far less frequently than hgcA and merA in most samples. Thus, the lack of merB to cleave the C–Hg bond suggests that demethylation was less likely to occur. 

**Figure 5:**
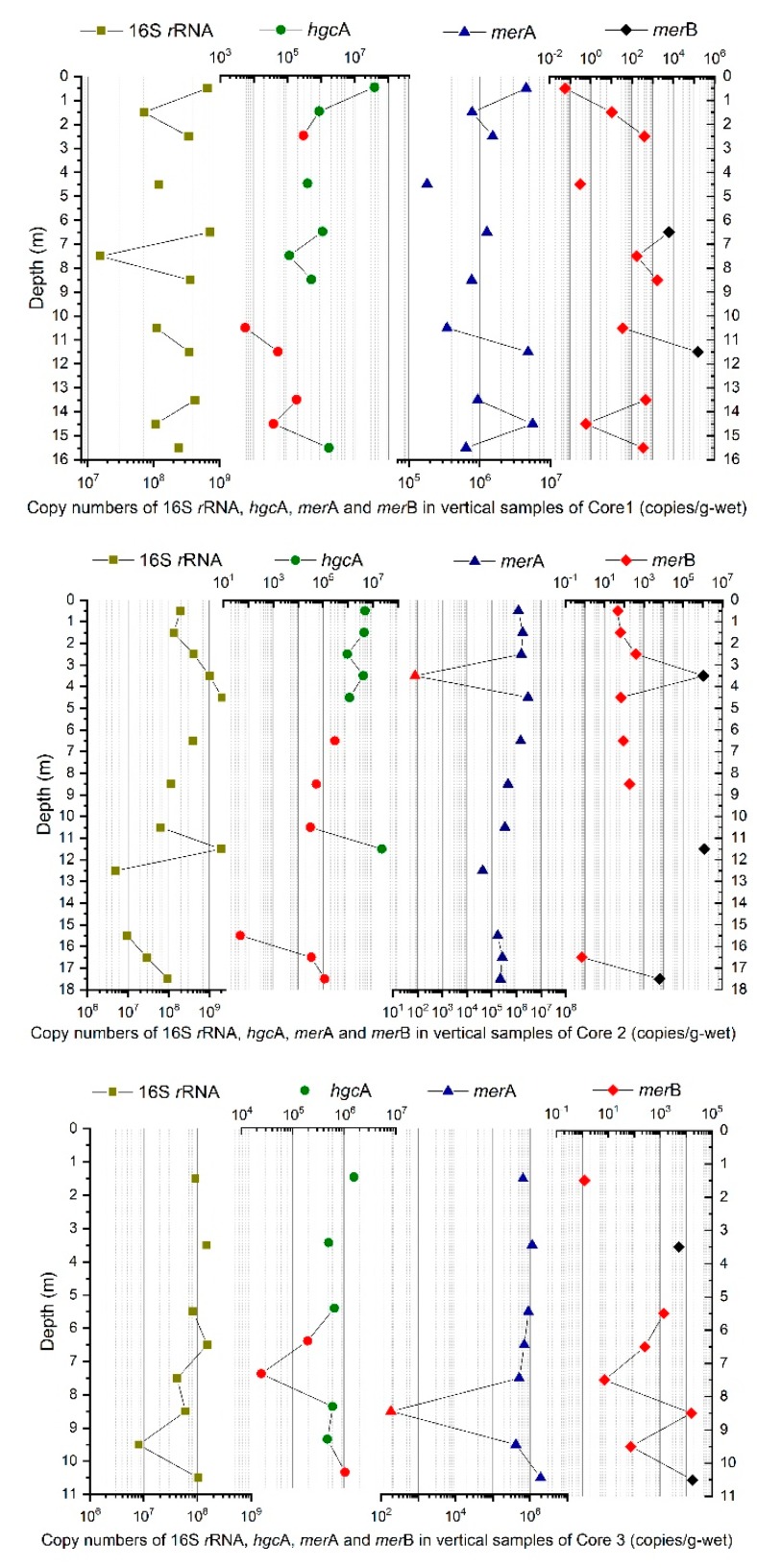
Transcripts abundance of 16S rRNA, hgcA, merA and merB in Core 1, 2 and 3; the red symbol indicates data that is below the detection limit, and that the results shown here are for reference.

**Figure 6:**
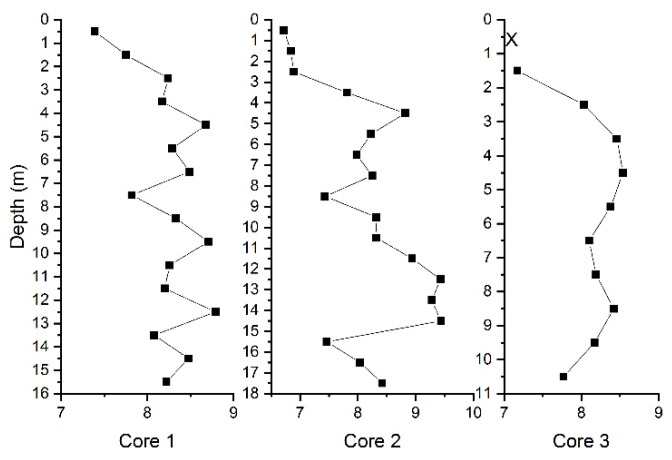
Vertical distribution of pH in core samples.

The transcripts abundance of 16S rRNA, hgcA, merA and merB in Core 1, 2 and 3 was shown in Figure 5. The high frequency of merA detection suggested a high potential for Hg^0^ generation, while merB was only present in the various points in the middle and bottom layers at orders of magnitude of 5–6. In anoxic sediments, a high Hg methylation potential is accompanied by a high demethylation potential in the same sediment [53]. However, in this case, expression of both hgcA and merA was abundantly detected only in the upper layers. The demethylation potential in the middle and lower layers was not equivalent to the methylation potential because of the lack of merB. In Core 3, hgcA was detected more frequently in the middle and lower layers, but the MeHg content was much lower than that at Core 1 and 2, also suggesting that the high levels of MeHg in Cores 1 and 2 may not be generated by the methylation process in the landfill. A significant correlation was found between THg and merA genes (r = −0.3745, *p* < 0.05), which indicated that merA led to more generation of Hg^0^, as well as gaseous Hg release, so that it caused THg loss in the samples. In addition, the alkaline pH of the soil (Figure 6 and Figure S1) hardly offers appropriate conditions for the bacteria to survive.

Considering both hgcA and merB, we suggest that biotic methylation took place in limited layers, and that the conditions in the landfill were not able to support biotic demethylation, indicating that biotic factors were not the dominant promoter to the methylation and demethylaiton processes. On the contrary, merA affected Hg reduction prevailingly, resulting in the production of Hg^0^. This is in accord with some studies about landfill gas emissions, suggesting that Hg^0^ accounted for more than 95% of total gaseous mercury [16,54].

## 4. Conclusions

The THg concentration ranged from 19 to 975 ng/g, with a mean value of 298 ng/g. In two samples, the ratio of the MeHg concentration to THg was between 15% and 20%, which was far higher than the normal level in soils or sediments. A significant correlation was observed between THg and MeHg in this study; however, we suggest more research on THg and MeHg in landfill sites to investigate the contribution of THg to MeHg. HgcA expression was abundant in the top and bottom layers, which indicates that waste inhibited the growth of bacteria. The merA gene was frequently detected in the same abundance as hgcA, and THg correlated with merA negatively. MerB was far less frequently detected in most samples, and only existed in middle or bottom layers of each core, which suggested demethylation process is hardly able take place in the landfill. More research is in needed to understand the behavior of inorganic and organic mercury in landfill sites.

## Figures and Tables

**Table 1:** THg concentration of core samples in each core.

Sample Number	Mean (ng/g)	Maximum (ng/g)	Minimum (ng/g)	Sd	Cv %
Core 1/16 (m)	226	975	19	224	99.11
Core 2/18 (m)	353	774	82	196	55.52
Core 3/11 (m)	316	552	58	164	51.90

**Table 2:** MeHg and its accumulation percentage of THg.

Sample Number	MeHg (ng/g)	THg (ng/g)					MeHg %
		n = 1	n = 2	n = 3	Mean	Sd	
Core 1/8 (m)	191	959	853	1113	975	131	19.6%
Core 1/11 (m)	5.3	327	350	354	344	15	1.5%
Core 2/3 (m)	100.7	880	533	418	610	241	16.5%
Core 2/12 (m)	13.9	793	617	556	655	123	2.1%
Core 3/4 (m)	3.2	541	789	324	551	233	0.6%
Core 3/9 (m)	8.7	226	689	295	403	250	2.2%

**Table 3:** 16S rRNA copy numbers in three cores.

Core	Number of Samples	Mean (Copies/g)	Maximum (Copies/g)	Minimum (Copies/g)
1	12	2.9 × 10^8^	7.1 × 10^8^	1.5 × 10^7^
2	13	5.4 × 10^8^	2.1 × 10^9^	5.0 × 10^6^
3	8	7.8 × 10^7^	1.5 × 10^8^	8.1 × 10^6^

**Table 4:** Correlations between THg, MeHg, MeHg/THg, hgcA, merA and merB.

Items	THg	MeHg	MeHg/THg	hgcA	merA	merB
THg	1					
MeHg	0.8553 *	1				
MeHg/THg	0.7539	0.9641 **	1			
hgcA	−0.0666	−0.2478	−0.2695	1		
merA	−0.3745 *	−1.734 × 10^−5^	0.1911	0.3036	1	
merB	0.2832	−0.2600	0.2897	0.3760 *	−0.1735	1

* *p* < 0.05; ** *p* < 0.01.

## Supplementary Materials

The following are available online at http://www.mdpi.com/1660-4601/15/6/1252/s1, Figure S1. pH value of samples in three cores; Table S1 Characteristics and compositions of waste in core samples; Table S2. Ratio of hgcA, merA and merB to 16S rRNA in three cores.
