# Supplementary material for: Vertical Distribution of Total Mercury and Mercury Methylation in a Landfill Site in Japan
Source: Int J Environ Res Public Health. 2018 Jun 13;15(6):1252. doi: 10.3390/ijerph15061252 (PMC6025181; doi:10.3390/ijerph15061252)
Supplement: Supplementary file 1 [file ijerph-15-01252-s001.pdf]

Table S1 Characteristics and compositions of waste in core samples

Sample number	color	speciaes	characteristic
Core 1-1	yellow light brown and little gray	loess	uniform and dry
Core 1-2	gray and yellow	clay	small block existing and compact
Core 1-3	black	fine,uniform sand	loose and little water
Core 1-4	brown	sand and stones	much water
Core 1-5	gray and concrete color	mud and small stones	much water
Core 1-6	gray and concrete color	small block	little water and compact
Core 1-7	dark black	coarse sand	broken plastics block and fibre
Core 1-8	gray and brown	unbreakable block	little plant
Core 1-9	black	sand	loose and uniform
Core 1-10	gray and concrete color	block and stones	solid
Core 1-11	gray	sand	loose
Core 1-12	gray	big stone and broken glass	much water
Core 1-13	gray	coarse sand and small sand	day and plastics bag existing
Core 1-14	black	mud and cotton-like	many plants and much water
Core 1-15	dark brown	only clay, no other object	loose and fine
Core 1-16	brown	sand	loose and little water
Core 2-1	brown	small stone and clay	solid block
Core 2-2	half gray and half brown	half clay and half sand	soft
Core 2-3	black (and white chemical)	fine sand	loose and plastics existing
Core 2-4	black	big stone and coarse sand	much water
Core 2-5	black	little soil and much plactics	much water
Core 2-6	black	coarse sand	much water and plactics bags existing
Core 2-7	dark gray	coarse sand	water existing and no ropiniss
Core 2-8	gray	coarse sand	loose and wire-like object existing

Core 2-9	dark gray	sand	loose and plants existing
Core 2-10	gray	small stone	loose, much water and plants existing
Core 2-11	black	stone and glass	wire and lamp of soil
Core 2-12	black	small stones	blue plastic bags and yellow plastic fragment
Core 2-13	gray and green	small stones and clay	plenty of plants
Core 2-14	brown	stone and clay	metal slice existing
Core 2-15	gray and yellow	many stones	humid
Core 2-16	black	small particle and no stone	uniform
Core 2-17	black to gray	humus	root and leaves existing
Core 2-18	yellow	all sand and small stones	much water and different size of stone
Core 3-1	black	small stones	plastic bags
Core 3-2	dark gray	very fine sand	uniform
Core 3-3	black	fine particles	no big object
Core 3-4	black and brown	sand existing and ropiness	broken glass
Core 3-5	black	clay	much water, fine and small quantity of grass
Core 3-6	black	clay	humid and fibre of plants existing
Core 3-7	black to gray	fine sand	withered grass and silver metal block
Core 3-8	black	humus	wood and fibre of plants existing
Core 3-9	yellow to golden	clay	solid and small stone and grass existing
Core 3-10	gray and yellow	stone and mud	much water
Core 3-11	brick color and dark red	sand	uniform,loose and much water

Table S2. Ratio of hgcA, merA and merB to 16S rRNA in three cores

Sample number	hgcA/16SrRNA	merA/16SrRNA	merB/16SrRNA
Core 1-1	5.85E-02	6.88E-03	8.91E-11
Core 1-2	1.23E-02	1.09E-02	1.48E-07
Core 1-3	8.80E-04	4.49E-03	1.21E-06
Core 1-4	-	-	-
Core 1-5	3.31E-03	1.52E-03	2.63E-09
Core 1-6	-	-	-
Core 1-7	1.56E-03	1.78E-03	8.70E-06
Core 1-8	7.41E-03	0.00E+00	1.13E-05
Core 1-9	1.42E-03	2.14E-03	4.62E-06
Core 1-10	-	-	-
Core 1-11	4.99E-05	3.13E-03	3.24E-07
Core 1-12	1.51E-04	1.38E-02	4.62E-04
Core 1-13	-	-	-
Core 1-14	4.40E-04	2.21E-03	1.10E-06
Core 1-15	3.54E-04	5.15E-02	5.52E-09
Core 1-16	7.09E-03	2.68E-03	1.48E-06
Core 2-1	2.49E-02	6.19E-03	2.45E-07
Core 2-2	3.32E-02	1.35E-02	4.86E-07
Core 2-3	2.29E-03	3.86E-03	9.67E-07
Core 2-4	4.11E-03	7.87E-08	1.07E-03
Core 2-5	5.50E-04	1.43E-03	3.29E-08
Core 2-6	-	-	-
Core 2-7	7.52E-04	3.77E-03	2.32E-07
Core 2-8	-	-	-
Core 2-9	4.69E-04	3.97E-03	1.63E-06
Core 2-10	-	-	-
Core 2-11	4.90E-04	5.45E-03	-
Core 2-12	1.15E-02	0.00E+00	6.20E-04
Core 2-13	-	8.62E-03	-
Core 2-14	-	-	-
Core 2-15	-	-	-
Core 2-16	5.08E-06	1.89E-02	-
Core 2-17	1.14E-03	9.18E-03	2.38E-08
Core 2-18	1.26E-03	2.43E-03	6.99E-05
Core 3-1	-	-	-
Core 3-2	1.70E-02	7.05E-03	1.34E-08
Core 3-3	-	-	-
Core 3-4	3.35E-03	7.60E-03	3.58E-05
Core 3-5	-	-	-
Core 3-6	7.81E-03	1.08E-02	1.67E-05
Core 3-7	1.28E-03	4.58E-03	1.67E-06
Core 3-8	5.85E-04	1.21E-02	1.71E-07

Core 3-9	1.00E-02	3.09E-06	2.61E-04
Core 3-10	5.87E-02	5.15E-02	9.15E-06
Core 3-11	9.99E-03	1.85E-02	1.71E-04

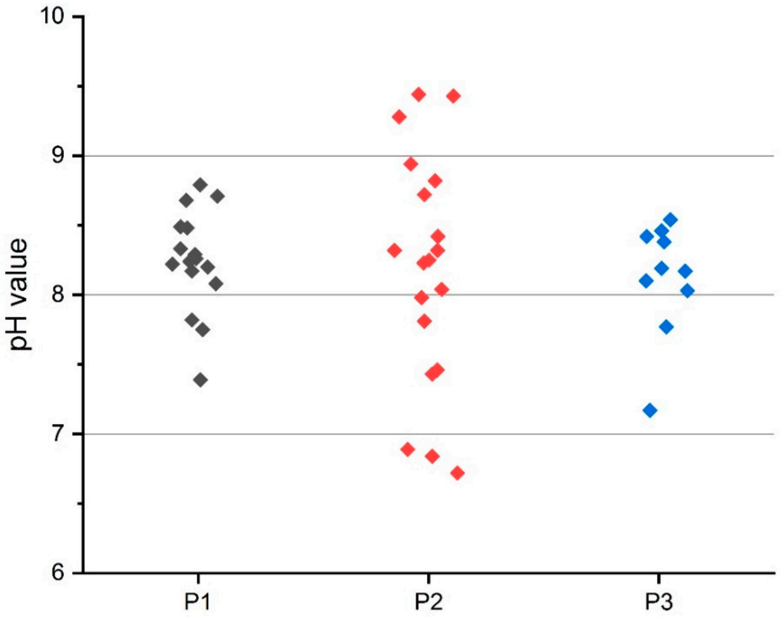

Figure S1. pH value of samples in three cores
